# Performance of multigene testing in cytologically indeterminate thyroid nodules and molecular risk stratification

**DOI:** 10.7717/peerj.16054

**Published:** 2023-09-18

**Authors:** Yuanyuan Zhou, Xinping Wu, Yuzhi Zhang, Zhiqiang Li, Xia Ge, Hao Chen, Yuan Mao, Wenbo Ding

**Affiliations:** 1 Genome Center, KingMed Center for Clinical Laboratory Co., Ltd, Hefei, Anhui Province, China; 2 Department of Ultrasound, Affiliated Hospital of Integrated Traditional Chinese and Western Medicine, Third Clinical Medical College, Nanjing University of Chinese Medicine, Nanjing, Jiangsu Province, China; 3 Department of Pathological Diagnosis, KingMed Center for Clinical Laboratory Co., Ltd, Hefei, Anhui Province, China

**Keywords:** Indeterminate cytology, Molecular testing, Molecular risk stratification, Next-generation sequencing, Thyroid nodule

## Abstract

**Objective:**

Thyroid cancer is the third most prevalent cancer among females. Genetic testing based on next-generation sequencing may provide an auxiliary diagnosis to reduce cytologically diagnostic uncertainty. However, commercial multigene tests are not widely available and are not well-tested in the Chinese population.

**Methods:**

In this study, we designed a multigene testing panel and evaluated its performance in 529 cytologically indeterminate thyroid nodules (Bethesda III, IV and V). The molecular data of the DNA mutations and RNA fusions of fine needle aspiration samples were reviewed in conjunction with a clinical diagnosis, pathological reports, and definitive surgery for retrospective analysis. Then, the molecular risk stratification was investigated for its accuracy in malignant risk prediction.

**Results:**

The overall combined consistency revealed substantial agreement (Kappa = 0.726) with the sensitivity, specificity, positive predictive value, and negative predictive values of 97.80%, 82.14%, 98.99%, and 67.65%, respectively. The most common aberration was *BRAF*^V600E^ (82.59%), followed by *NRAS* mutants (4.07%), *RET* fusions (3.70%), and *KRAS* mutants (3.15%). Two cases (0.44%) were categorized into a high-risk group, 426 cases (94.67%) were categorized into a *BRAF*-like group with totally histopathologic papillary patterned tumors, and 22 cases (4.89%) were categorized into a *RAS*-like group with 14 papillary and eight follicular patterned tumors when the cohort concurrent aberrations were excluded. Potentially aggressive features may be related to concurrent molecular alterations of *BRAF*^V600E^ with *TERT*^Q302R^, and *AKT1*^L52R^, *NRAS*^G12C^, *NRAS*^Q61R^, and *CCDC6-RET* fusions.

**Conclusions:**

This study provided a multigene panel for identifying benign nodules from cytologically indeterminate thyroid nodules to avoid unnecessary surgery. We provide further evidence for using molecular risk stratification as a promising predictor of disease outcomes. The results of this study may be limited by the extremely high prevalence of cancer in the cohort for clinical reference.

## Introduction

Thyroid cancer was the third highest prevalent cancer among females in 2022. Its incidence has increased dramatically but its mortality rate has remained low in both the United States and China since 2000 (Miller et al., 2022; Xia et al., 2022). As socioeconomic levels and radiologic technology have improved, the incidence rate of thyroid cancer has increased in transitioned countries over that in transitioning countries. This increase may be attributable to overdiagnosis (*e.g.*, ionizing radiation), obesity, and exposure to hormones and environmental pollutants, *etc*. (Sung et al., 2021). The active surveillance for microcarcinoma (*e.g.*, optimizing molecular markers and improved risk stratification) is recommended in place of frequent imaging screenings (Sung et al., 2021) due to the indolent property of small thyroid tumors.

Fine needle aspiration cytology (FNAC) is the gold standard technique for thyroid nodules with suspicious ultrasound features. However, its accuracy is limited in cytologically indeterminate samples (Ulisse et al., 2021). According to the latest version of The Bethesda System for Reporting Thyroid Cytopathology (TBSRTC), 20%∼30% of nodules may be defined as having indeterminate cytology with the risk of malignancy ranging from 10%∼75%. There are three categories of classification for these malignancies; these include the Bethesda category III (atypia of undetermined significance or follicular lesion of undetermined significance, AUS/FLUS), Bethesda category IV (follicular neoplasm/ suspicious for a follicular neoplasia, SFN/FN), and Bethesda category V (suspicious for malignancy, SFM) (Baloch et al., 2018). Pathologists tend toward the conservative management of this disease, lending itself to conflicts in the physician-patient relationship and moderate reproducibility of cytologically indeterminate samples (Sauter et al., 2019). Furthermore, the performance of interventional radiologists in providing ultrasound-guided thyroid fine needle aspirations (FNAs) and the experience of endocrinologists and cytopathologists in interpreting aspirates varies among institutions or hospitals (Sauter et al., 2019; Bose, Sacks & Walts, 2019). These may result in repeat FNA, diagnostic lobectomy, and even unnecessary total thyroidectomy procedures. Thus, molecular pathogenesis has become a promising approach to supplement cytologic examination for preoperative diagnosis and treatment options (Ren et al., 2022; Lee et al., 2022).

Diverse molecular testing panels have been developed based on next-generation sequencing (NGS) techniques. For instance, ThyroSeq, ThyGenX and ThyraMIR tests have both high PPV and NPV and can be used to determine malignancy, while Afirma GSC tests show high NPV and relatively low PPV and might be useful to rule-out the presence of malignancy for indeterminate thyroid lesions (Bose, Sacks & Walts, 2019; Lee et al., 2022; Rossi et al., 2022). There is limited research applying these tests in the Chinese population and few verified self-designed multigene panels are available on the market (Ren et al., 2022; Song et al., 2020). Here, we designed a multigene NGS panel to detect targeted DNA alterations and RNA fusions. We retrospectively evaluated its performance in FNA specimens of cytologically indeterminate thyroid nodules and compared these results to those of a clinical diagnosis. Our study proposed an auxiliary diagnosis to reduce cytologically diagnostic uncertainty and to explore the possible application of molecular risk stratification based on our NGS panel. Furthermore, this study isolated nodules with concurrent molecular alterations from single aberrations and analyzed the two sets of data separately.

## Materials & Methods

### Study cohorts and sample collection

**Figure 1:**
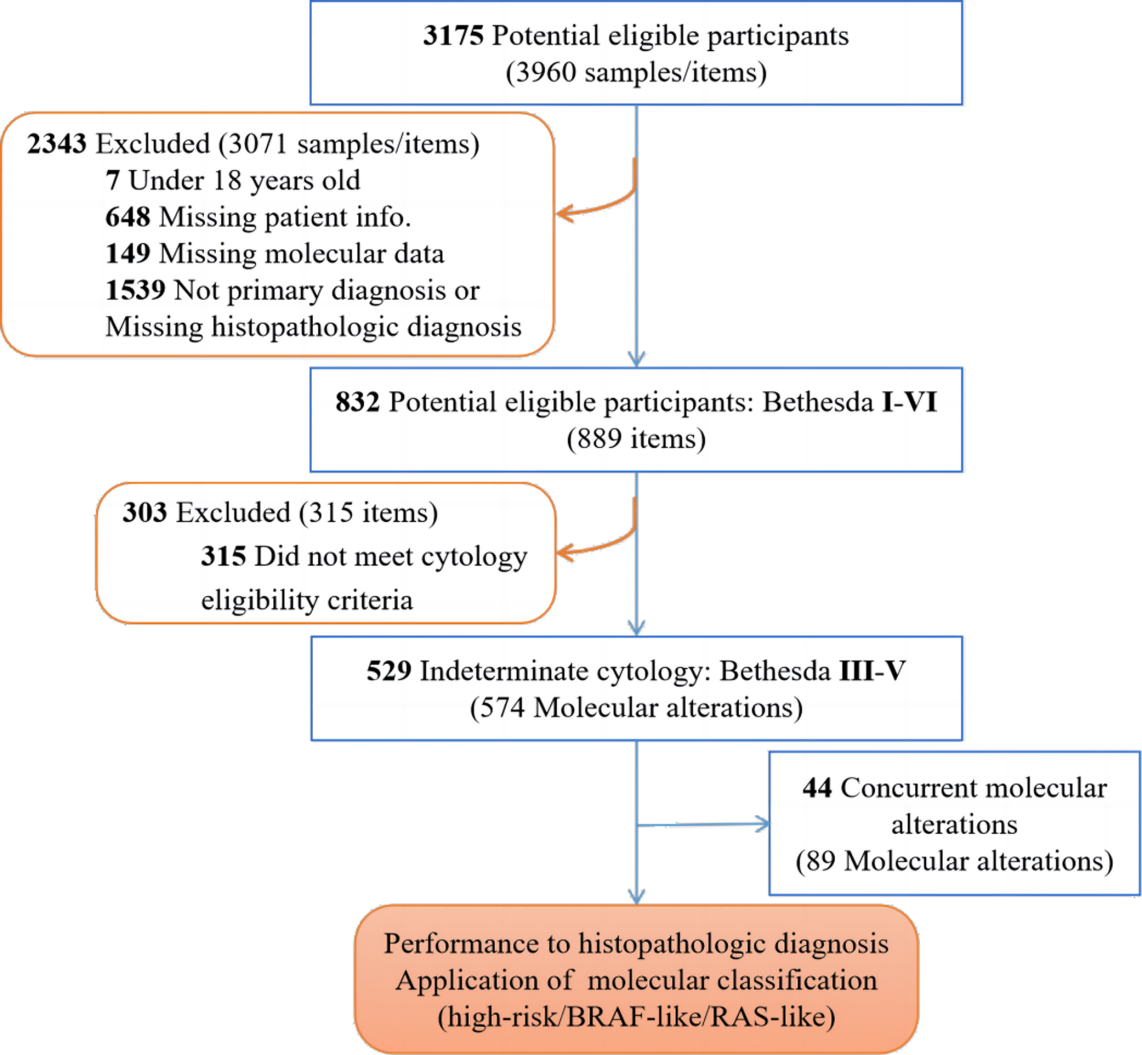
Recruitment and exclusion of patients and samples in the study.

Patients with at least one thyroid nodule that was clinically diagnosed and confirmed by ultrasound were retrospectively enrolled and screened in our study (3,175 cases). Patients under 18 years old, who did not have a primary diagnosis, or who were missing the required information (2,343 cases) were excluded, as detailed in Fig. 1. Fine-needle aspiration (FNA) samples were collected under ultrasound guidance by radiologists using a 22-gauge needle. The aspirate was smeared on microscope slides and stained after being fixed with 95% ethanol for cytological examination. Categorization was conducted according to The Bethesda System for Reporting Thyroid Cytopathology (TBSRTC). A total of 529 FNA samples from cytologically indeterminate thyroid nodules (*i.e.,* diagnosed by clinical pathologists as Bethesda III, IV or V) were collected to analyze the DNA mutations and RNA fusions *via* NGS. The clinical diagnosis of the nodules and their subsequent follow-up reports following definitive surgery, if performed (Table 1) were analyzed retrospectively for further investigation. This retrospective study was approved by the Ethics Committee of Jiangsu Integrated Traditional Chinese and Western Medicine Hospital (No. 2022-LWKY-042) and the Ethics Committee of Guangzhou KingMed Medical Laboratory Center (No. 2023005). Patient consent was waived due to the observational nature of the retrospective study and patient identities were kept anonymous (No. 2023005).

**Table 1:** Baseline characteristics of thyroid nodules with molecular pathology results (*n*, column %).

**Characteristics**	**Molecular pathology**		*χ* ^2^	***P*** **-value**
	**Benign nodules (*n* = 34)**	**Suspicious nodules (*n* = 495)**		
Age at diagnosis				
Under 55 years	21 (61.76)	403 (81.41)	7.721	0.005
55 years and older	13 (38.24)	92 (18.59)		
Gender				
Female	28 (82.35)	391 (78.99)	0.218	0.64
Male	6 (17.65)	104 (21.01)		
Cytologic classification				
Bethesda III nodules	6 (17.65)	71 (14.34)	38.829	<0.001
Bethesda IV nodules	12 (35.29)	30 (6.06)		
Bethesda V nodules	16 (47.06)	394 (79.60)		
Thyroid surgery				
Surgery	30 (88.24)	488 (98.59)	45.762	<0.001
No surgery/Unknown	4 (11.76)	7 (1.41)		

### Molecular analysis

Sequencing libraries were generated using one-step multiplex PCR targeted amplicons. Briefly, the genomic DNA were isolated from the FNA samples of thyroid nodules using the QIAamp DNA Mini kit (Qiagen, Hilden, Germany) in accordance with the manufacturer’s specifications. The concentrations were determined by Qubit 3.0 Fluorometer (Thermo Fisher Scientific, Waltham, MA, USA). Sample DNA were mixed with KAPA2G Fast PCR (Roche, La Posay, France) and were amplified to detect driver gene variants, utilizing our multigene testing panel (Table S1) for the detection of 14 thyroid cancer-related genes (*AKT1, BRAF, CTNNB1, EIF1AX, HRAS, KRAS, NRAS, PAX8, PIK3CA, PTEN, RET, TERT, THADA,* and *TP53*) and 21 types of gene rearrangements occurring in thyroid cancer (*ACBD5, AFAP1L2, ALK, ATG10, BRAF, CALM2, CCDC6, ERC1, ETV6, FLNC, FMNL2, KIAA1217, KIAA1594, KIF20B, NCOA4, NTRK3, PAK1, PAX8, PIBF1, PPAR γ, PXK, RALGAPA2, RET, SND1,* and *STRN*). Superfluous primers were purified using Agencourt AMPure XP 60 mL kit (Beckman, Brea, CA, USA). The concentrations of barcoded PCR produced library were then measured and diluted to 100 pM. A total of 20 µL pooled amplicons were sequenced at the Ion Proton system (Thermo Fisher Scientific, Waltham, MA, USA). Local alignments of reads to the hg19 genome were performed *via* Bowtie2 (version 2.2.4) in the paired-end mode. SAM alignment files were converted to BAM files, sorted, and indexed using Samtools (version 0.1.19), following by a procession with Bam-read count and a customized written Perl script.

### Test performance evaluation and false-positive/-negative (FP/FN) results analysis

The actual status of each diagnosis was determined: (1) by surgical pathology or (2) a benign molecular test without surgical pathology. The results of the benign molecular test are only considered truly benign when they are based on ultrasound and/or clinical characteristics. Test performance characteristics including sensitivity, specificity, positive predictive value (PPV), negative positive predictive value (NPV), and Kappa-value for consistency check were calculated at 95% confidence interval (CI) for the overall cytologically indeterminate specimens, as well as separately for nodules in Bethesda III, IV and V following the established method (Altman & Bland, 1994). Based on observed sensitivity and specificity, hypothetical PPV and NPV curves were modeled over the entire range of possible disease prevalence (0–100%), allowing the observed and the anticipated PPV and NPV to be compared. In order to preclude misdiagnosis due to false-negative test results, histologic slides of nodules that had negative molecular results on FNA cytology but had been diagnosed as malignant on resection, were blindly reviewed by another pathologist.

### Molecular risk stratification

Thyroid nodules with molecular aberrations were categorized into three molecular risk groups (MRGs): a high-risk group (*TERT* or *TP53* alterations), a *BRAF*-like group (mainly *BRAF*^V 600E^) and a *RAS*-like group (*HRAS, KRAS, NRAS,* and others). The classification of all variants into this three-category system is shown in Table S2. A sample would be considered positive for molecular pathology if some genetic alteration was detected; the sample would be negative if no variants were detected in our panel. The MRG results would be assessed for the relevance with clinicopathologic diagnosis and further analyzed for the aggressive features of tumor-node-metastasis (TNM) staging. Nodules with concurrent molecular alterations were isolated and their distribution in MRGs and their TNM classification were assessed individually. Slides of surgical histopathology for special cases (nodules suspected as false-negative or false-positive, presenting concurrent molecular alterations, and other infrequent genetic alterations) were retrospectively analyzed by another pathologist.

### Statistical analyses

Descriptive summaries of the histopathology and molecular pathology reports were shown with counts and percentages. Pearson chi-square tests were used to compare the categorical variables of age, gender, Bethesda category, and thyroid surgery between benign/suspicious molecular result groups. Statistical analysis was conducted utilizing SPSS software (version 26.0) with *P*-value < 0.05 being considered as statistically significant.

## Results

### Study cohort and demographics

A total of 529 cases with cytologically indeterminate thyroid FNA results were identified between February 2021 to December 2021 at Jiangsu Province Hospital on Integration of Chinese and Western medicine in China. These cases were analyzed for molecular testing *via* NGS. All data in the cohort are accessible in Table S2. The patient characteristics and thyroid nodules are shown in Table 1. A total of 391 (79.88%) patients with suspicious nodules as determined by molecular tests were female and 403 (81.41%) of the cases for the cohort were below 55 years old at time of diagnosis. The majority of thyroid nodules (410 cases, 77.50%) were interpreted as Bethesda V, and 14.56% (77 cases), and 7.94% (42 cases) were Bethesda III and IV, respectively. Overall, the results of our thyroid NGS panel determined benign nodules (34 cases, 6.43%) and suspicious nodules (495 cases, 93.57%). The accuracy of the test was checked to evaluate the performance of the multigene panel. Furthermore, 574 molecular aberrations with 89 samples containing coexisting nodules were further investigated for their associated histopathologic cancer types and TNM staging based on molecular risk stratification (Fig. 1).

### Test performance

**Figure 2:**
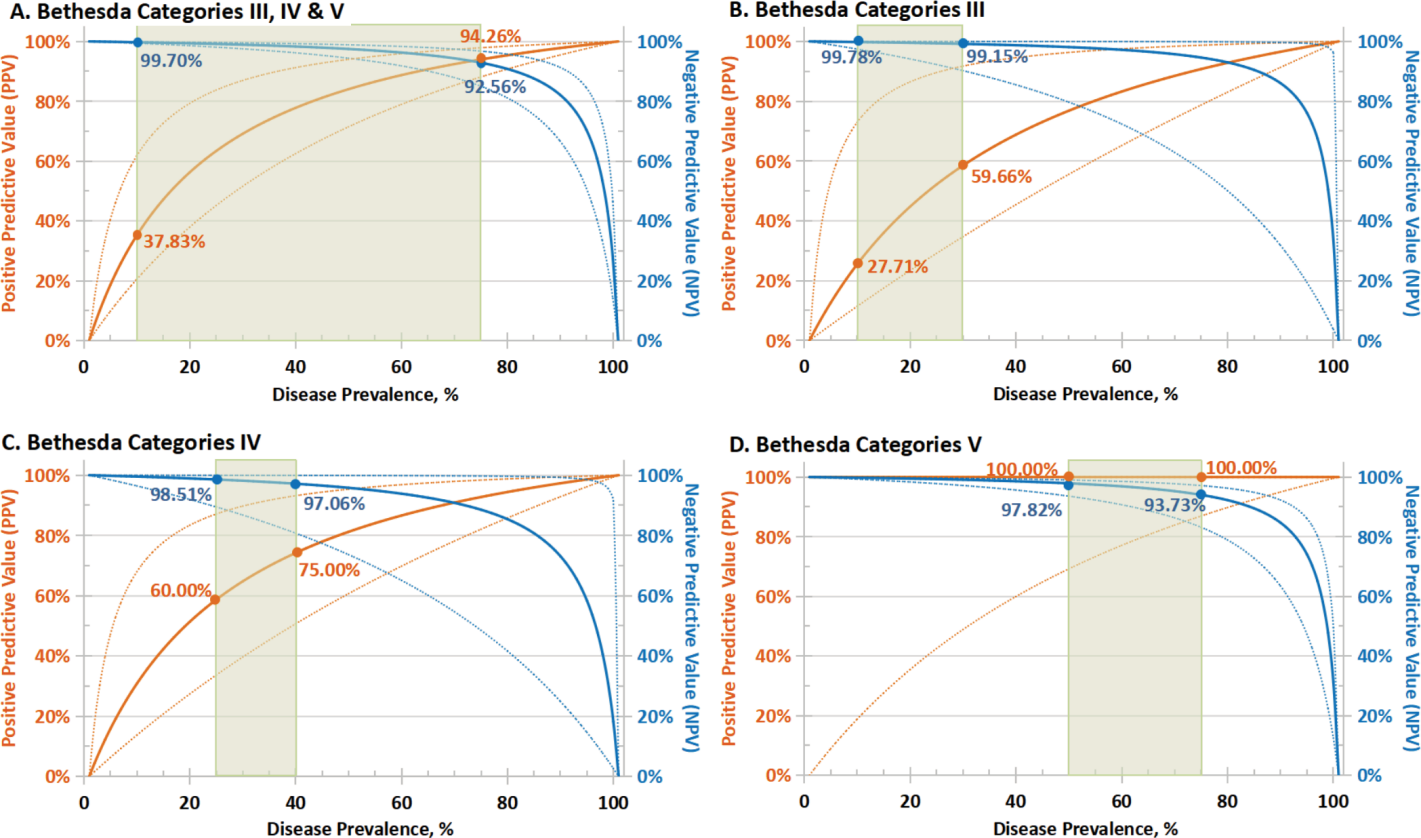
Predicated performance of molecular test in populations with different disease prevalence. Predicated PPV (solid orange lines) and NPV (solid blue lines) with 95% CI (dotted lines) based on sensitivity and specificity for: (A) the overall cytologically indeterminate specimens (Bethesda III, IV and V); (B–D) Bethesda III, IV and IV cytology thyroid nodules separately. NPV and PPV in the expected range of cancer/NIFTP prevalence (green rectangle) based on the malignant risk of different categories by Bethesda system were shown. PPV, positive predictive value; NPV, negative predictive value; CI, confidence interval; NIFTP, noninvasive follicular thyroid neoplasm with papillary-like nuclear features.

The test performance for sensitivity, specificity, NPV, PPV, and consistency of the cytologic groups of thyroid nodules is presented in Table 2. Since sensitivity and specificity are intrinsic characteristics for each test, the NPV and PPV depended on the prevalence of the disease in the screened population (Smith, 2012). Predicated PPV and NPV with 95% CI were calculated based on the observed sensitivity and specificity; hypothetical PPV and NPV curves were modeled over the entire range of possible disease prevalence (0–100%), as depicted in Fig. 2.

**Table 2:** Performance of the molecular test in cytologically indeterminate thyroid nodules.

**Performance in Bethesda III nodules (*n* = 77; disease prevalence 90.91%)**					
Molecular test	Histopathologic diagnosis		Test performance, % (95% CI)		Consistency check
	Positive (*n* = 70)	Negative (*n* = 7)			
Positive	69	2	PPV, 97.18 (91.45–99.11)	Sensitivity, 98.57 (92.30–99.96)	Kappa = 0.748
Negative	1	5	NPV, 83.33 (40.32–97.37)	Specificity, 71.43 (29.04–96.33)	Substantial agreement
**Performance in Bethesda IV nodules (*n* = 42; disease prevalence 66.67%)**					
Molecular test	Histopathologic diagnosis		Test performance, % (95% CI)		Consistency check
	Positive (*n* = 28)	Negative (*n* = 14)			
Positive	27	3	PPV, 90.00 (76.70–96.09)	Sensitivity, 96.43 (81.65–99.91)	Kappa = 0.778
Negative	1	11	NPV, 91.67 (61.16–98.72)	Specificity, 78.57 (49.20–95.34)	Substantial agreement
**Performance in Bethesda V nodules (*n* = 410; disease prevalence 98.29%)**					
Molecular test	Histopathologic diagnosis		Test performance, % (95% CI)		Consistency check
	Positive (*n* = 403)	Negative (*n* = 7)			
Positive	394	0	PPV, 100.00 (–)	Sensitivity, 97.77 (95.80–98.97)	Kappa = 0.599
Negative	9	7	NPV, 43.75 (28.96–59.74)	Specificity, 100.00 (59.04–100.00)	Moderate agreement
**Performance in Bethesda III, IV and V nodules (*n* = 529; disease prevalence 94.71%)**					
Molecular test	Histopathologic diagnosis		Test performance, % (95% CI)		Consistency check
	Positive (*n* = 501)	Negative (*n* = 28)			
Positive	490	5	PPV, 98.99 (97.79–99.54)	Sensitivity, 97.80 (96.11–98.90)	Kappa = 0.726
Negative	11	23	NPV, 67.65 (53.20–79.36)	Specificity, 82.14 (63.11–93.94)	Substantial agreement

Among the positive test samples, 490 cases (98.99%) were malignant and five cases (1.01%) were benign on surgery resection, while 23 cases (67.65%) were benign and 11 cases (32.35%) were cancerous within the negative test samples. Overall, in the cohort of cytologically indeterminate nodules had a cancer prevalence of 94.71%; multigene testing presented a sensitivity of 97.80% (95% CI [96.11–98.90]%), a specificity of 82.14% (95% CI [63.11–93.94]%), a PPV of 98.99% (95% CI [97.79–99.54]%) and an NPV of 67.65% (95% CI [53.20–79.36]%) (Table 2). The tumor prevalence in our cohort was obviously higher than clinical actuality due to the difficulty of identifying “true” benign nodules without surgery, risks of malignancy (with NIFTP) published in the TBSRTC diagnostic categories were referenced as the expected prevalence range (marked with green rectangles in Fig. 2). Moreover, the consistency of the multigene testing performed better in Bethesda III and IV (Kappa = 0.748 and 0.778, respectively, ranked as substantial agreement) than V (Kappa = 0.599, ranked as moderate agreement) specimens. When three categories of cytologically intermediate nodules were pooled, the consistency check for molecular test with histopathologic diagnosis manifested substantial agreement (Kappa = 0.726) (Table 2).

### Summary of molecular alterations

All the somatic single nucleotide variants (SSNVs), insertions, deletions, duplications, and fusions detected in cytological indeterminate thyroid nodules were summarized in Table 3. The most observed gene aberration in this cohort was *BRAF*^V 600E^ (446 cases, 82.59%), followed by *RET* aberrations (20 fusions and two mutants, 4.07%), *NRAS* mutants (22 cases, 4.07%), *KRAS* mutants (17 cases, 3.15%), and other alterations. Notably, *CCDC6-RET* fusion was detected in 17 cases, which may be related to 16 patients with papillary thyroid carcinoma or microcarcinoma (PTC/PTMC) and one benign case with concurrent molecular alteration. Moreover, some molecular aberrations may co-exist with others rather than appear alone; these include *BRAF* rearrangements and deletions, *PTEN* deletions, *TERT*^Q302R^, and mutants of *AKT1*, *EIF1AX*, *TP53,* and *PIK3CA*. There are some molecular alterations that had never been reported in solid tumors before, including *BRAF-PXK* fusion, *FLNC-BRAF* fusion, *BRAF*^N486_T491delinsS^, *PAX8*^C147R^, *PTEN*^D252Rfs∗42^, and *TERT*^Q302R^, as well as the *EIF1AX* c.338-2A>T splice site mutation and the *KIAA1217-RET* fusion in thyroid cancers.

**Table 3:** Summary of molecular alterations in cytologically indeterminate thyroid nodules.

**Gene**	**Variant** ^**a**^	**Alteration (Count)**	**Count**	**% of Total**	**Referene**
**AKT1**	SSNV	exon4 NM_001014432.1: c.155T>G (p.L52R)	2	0.37%	
	SSNV	exon4 NM_001014432.1: c.49G>A (p.E17K)	1	0.19%	
**ALK**	fusion	STRN-ALK	1	0.19%	Chu et al. (2020)
**BRAF**	SSNV	exon15 NM_004333.4: c.1799T>A (p.V600E)	446	82.59%	
	SSNV	exon15 NM_004333.4: c.1801A>G (p.K601E)	1	0.19%	
	indel	exon12 NM_004333.4: c.1456_1471delinsT (p.N486_T491delinsS)	1	0.19%	
	focal deletion	exon12 NM_004333.4: c.1457_1471del (p.N486_P490del)	1	0.19%	
	fusion	BRAF-PXK	1	0.19%	
	fusion	FLNC-BRAF	1	0.19%	
	fusion	SND1-BRAF	1	0.19%	Chu et al. (2020)
**EIF1AX**	SSNV	exon6 NM_001412.3: c.338-2A>T (p.?)	1	0.19%	Castagna et al. (2020)
**HRAS**	SSNV	exon2 NM_005343.2: c.34G>A (p.G12S)	1	0.19%	
	SSNV	exon3 NM_005343.2: c.181C>A (p.Q61K)	1	0.19%	
	SSNV	exon3 NM_005343.2: c.182A>G (p.Q61R)	1	0.19%	
	SSNV	exon3 NM_005343.2: c.182A>T (p.Q61L)	1	0.19%	
**KRAS**	SSNV	exon3 NM_004985.3: c.181C>A (p.Q61K)	4	3.15%	
	SSNV	exon2 NM_004985.3: c.35G>A (p.G12D)	3	0.56%	
	SSNV	exon2 NM_004985.3: c.35G>T (p.G12V)	3	0.56%	
	SSNV	exon2 NM_004985.3: c.38G>A (p.G13D)	2	0.37%	
	SSNV	exon3 NM_004985.3: c.182A>G (p.Q61R)	1	0.19%	
	SSNV	exon4 NM_004985.3: c.436G>A (p.A146T)	1	0.19%	
	SSNV	exon4 NM_004985.3: c.437C>T (p.A146V)	1	0.19%	
	SSNV	exon2 NM_004985.3: c.34G>A (p.G12S)	1	0.19%	
	SSNV	exon2 NM_004985.3: c.37G>C (p.G13R)	1	0.19%	
**NRAS**	SSNV	exon3 NM_002524.4: c.182A>G (p.Q61R)	17	3.15%	
	SSNV	exon2 NM_002524.4: c.35G>A (p.G12D)	2	0.37%	
	SSNV	exon3 NM_002524.4: c.181C>A (p.Q61K)	2	0.37%	
	SSNV	exon2 NM_002524.4: c.34G>T (p.G12C)	1	0.19%	
**PAX8**	SSNV	exon5 NM_003466.3: c.439T>C (p.C147R)	1	0.19%	
	SSNV	exon7 NM_003466.3: c.659G>A (p.R220Q)	1	0.19%	
**PIK3CA**	SSNV	exon10 NM_006218.2: c.1624G>A (p.E542K)	1	0.19%	
	SSNV	exon10 NM_006218.2: c.1633G>A (p.E545K)	1	0.19%	
	SSNV	exon12 NM_006218.2: c.1850G>A (p.R617Q)	1	0.19%	
	SSNV	exon21 NM_006218.2: c.3140A>G (p.H1047R)	1	0.19%	
	SSNV	exon21 NM_006218.2: c.3140A>T (p.H1047L)	1	0.19%	
**PTEN**	SSNV	exon5 NM_000314.4: c.323T>C (p.L108P)	1	0.19%	
	focal deletion	exon5 NM_000314.4: c.437del (p.L146*)	1	0.19%	
	focal deletion	exon7 NM_000314.4: c.754_764del (p.D252Rfs*42)	1	0.19%	
**RET**	fusion	CCDC6-RET	17	3.15%	Chu et al. (2020) Lee et al. (2016)
	fusion	NCOA4-RET	2	0.37%	
	fusion	KIAA1217-RET	1	0.19%	
	SSNV	exon16 NM_020975.4: c.2753T>C (p.M918T)	1	0.19%	Subbiah et al. (2021); Ghazani et al. (2020); Alzahrani et al. (2022)
	SSNV	exon14 NM_020975.4: c.2410G>A (p.V804M)	1	0.19%	
**TERT**	SSNV	exon2 NM_198253.2: c.905A>G (p.Q302R)	1	0.19%	
**TP53**	SSNV	exon10 NM_000546.5: c.1073A>T (p.E358V)	1	0.19%	
	SSNV	exon7 NM_000546.5: c.731G>A (p.G244D)	1	0.19%	
	SSNV	exon7 NM_000546.5: c.734G>T (p.G245V)	1	0.19%	
	SSNV	exon7 NM_000546.5: c.742C>T (p.R248W)	1	0.19%	
	focal deletion	exon4 NM_000546.5: c.245_246del (p.P82Rfs*66)	1	0.19%	
	focal deletion	exon8 NM_000546.5: c.783-806del (p.G262-S269del)	1	0.19%	
	duplication	exon4 NM_000546.5: c.273dup (p.P92Afs*57)	1	0.19%	
**Total**			540	100%	

### False-negative and false-positive molecular test results with histopathologic diagnosis

The clinicopathologic characteristics of 11 false-negative (Case 1 to Case 11) and five false-positive (Case 12 to Case 16) cases are listed in Table 4. In total, 11 malignant nodules tested negative in our cohort, including one in Bethesda III, one in Bethesda IV, and nine in Bethesda V. These patients underwent surgery due to other suspicious features, subject to their preference. All of the thyroid nodules proved to be PTC/PTMC after diagnostic surgery, in which the presence of extrathyroidal invasion (T4a_staging) was observed in three cases. Four cases revealed locoregional nodal metastases (N1a/b_staging). Two nodules that tested negative before surgery (Case 1 and Case 4) were verified positive with the *BRAF*^V 600E^ mutant on resection, indicating that molecular misdiagnosis may be the result of a limited tumor volume, sampling technique, or preservation method.

**Table 4:** Clinicopathologic characteristics of cases with false-positive (FP) or false-negative (FN) test results.

**Case No.**	**Age (year)**	**Gender (M/F)**	**Nodule size (cm)** ^a^	**Cytology diagnosis**	**Histological diagnosis**	**T_stage**	**N_stage**	**Molecular aberrations**	**Other indicators**	**Disagreement**
1	63	F	1.3	Bethesda V	PTC	T1b	N0	Undetected	ARMS-PCR: *BRAF*^V 600E^ (+)	FN
2	50	F	1.1	Bethesda V	PTC	T1b	N1a	Undetected	–	FN
3	55	F	1.0	Bethesda V	PTC	T1a	N1a	Undetected	–	FN
4	46	F	0.7	Bethesda IV	PTMC	T1a	N0	Undetected	ARMS-PCR: *BRAF*^V 600E^ (+)	FN
5	66	F	0.7	Bethesda V	PTMC; Lymphocytic thyroiditis	T4a	N1b	Undetected	LN-FNA-T *g* > 500.000 ng/mL	FN
6	22	F	0.7	Bethesda V	PTMC	T4a	N0	Undetected	–	FN
7	50	F	0.6	Bethesda III	PTMC	T1a	N1b	Undetected	–	FN
8	48	M	0.5	Bethesda V	PTMC	T1a	N0	Undetected	–	FN
9	45	F	0.5	Bethesda V	PTMC	T4a	N0	Undetected	–	FN
10	40	F	0.5	Bethesda V	PTMC	T1a	N0	Undetected	–	FN
11	53	F	0.3	Bethesda V	PTMC	T1a	Nx	Undetected	–	FN
12	37	F	3.0	Bethesda IV	Follicular adenoma (FA)	NA	NA	*BRAF* ^V 600E^	NA	FP
13	62	M	NA	Bethesda III	Lymphocytic thyroiditis	NA	NA	*BRAF* ^V 600E^	NA	FP
14	49	F	NA	Bethesda III	Nodular goiter with adenomatoid nodule	NA	NA	*BRAF* ^V 600E^	NA	FP
15	55	F	NA	Bethesda IV	Nodular goiter with adenomatoid nodule; Lymphocytic thyroiditis	NA	NA	*NRAS*^Q61R^; *KRAS*^A146V^	NA	FP
16	53	F	NA	Bethesda IV	Hashimoto’s thyroiditis (HT) with adenomatoid nodule	NA	NA	*BRAF*^V 600E^; *CCDC6-RET*	NA	FP

**Notes.**

a Measured in pathological specimen.

The false-negative result of Case 5 may be specious, since the value of FNA-Tg was significantly high (above 500.000 ng/mL) in the puncture fluid of the left lymph nodule and FNAC manifested chronic lymphocytic thyroiditis. However, none of the molecular aberrations were detected in either the thyroid and lymph nodules at diagnosis. Similarly, the false-positive result of Case 12 may require further study for a potentially malignant transformation, given that the final histological diagnosis was benign follicular adenoma (FA), three cm in diameter. This nodule was suspected as being follicular neoplasia by FNA cytology and the *BRAF*^V 600E^ mutant was detectable before surgery.

### Molecular risk stratification (concurrent molecular alterations excluded)

The cancer subtypes in specific molecular alterations and MRGs are summarized in Table 5. After the exclusion of concurrent molecular alterations, only two nodules with TP53 alterations were divided into the high-risk group, one (*TP53*^P82Rfs∗66^) of which was follicular thyroid carcinoma (FTC) with partial differentiation. The other (*TP53*^G262−S269del^) was determined to be PTMC on resection, however it was initially cytologically categorized as Bethesda IV (suspicious for a follicular neoplasia). The prevalence of PTC/PTMC in the *BRAF*-like group reached 100% in 426 tumors, while 22 malignant nodules in the *RAS*-like group were composed of 14 PTC/PTMC (63.64%) and eight FTC or follicular patterned tumors (follicular variant PTC/PTMC, FV-PTC/FV-PTMC) (36.36%).

**Table 5:** Probability of cancer in specific molecular alterations and classifications (concurrent molecular alterations excluded).

**Molecular classification**	**Prevalence in test-positive samples, No. (%)**	**Molecular alterations, No.**		**Histopathologic diagnosis, No.**						**Cancer type**	**Cancer type, No. (%)**
				**Benign**	**FTC**	**FV-PTMC**	**FV-PTC**	**PTMC**	**PTC**		
High-risk group	2 (0.44)									PTC/PTMC	1 (50)
		TP53	2	0	1	0	0	1	0	FV-PTC/FV-PTMC/FTC	1 (50)
BRAF-like group	426 (94.67)										
		V600E-BRAF	407	3	0	0	0	135	269	PTC/PTMC	423(100)
		CCDC6-RET	15	0	0	0	0	4	11	FV-PTC/FV-PTMC/FTC	0 (0)
		KIAA1217-RET	1	0	0	0	0	1	0		
		NCOA4-RET	2	0	0	0	0	0	2		
		RET	1	0	0	0	0	1	0		
RAS-like group	22 (4.89)										
		non-V600E-BRAF	2	0	0	0	0	0	2	PTC/PTMC	14 (63.64)
		HRAS	1	0	0	0	0	0	1	FV-PTC/FV-PTMC/FTC	8 (36.36)
		KRAS	6	0	0	0	0	0	6		
		NRAS	9	0	3	2	1	0	3		
		PAX8	2	0	0	1	0	1	0		
		PTEN	1	0	1	0	0	0	0		
		STRN-ALK	1	0	0	0	0	1	0		
Undetected	NA									PTC/PTMC	11 (100)
		—	34	23	0	0	0	9	2	FV-PTC/FV-PTMC/FTC	0 (0)
Total	450 (100)		484	26	5	3	1	153	296		

### TNM staging and aggressive features (concurrent molecular alterations excluded)

The extrathyroidal extension and TNM stage of thyroid carcinomas were proposed to be associated with molecular risk subtyping (Hong et al., 2022). Thus, we investigated the aggressive features of the molecular alterations *via* the categorization of MRGs in 485 nodules with known TNM staging information (data displayed in Table S2). Among 174 *BRAF*-like tumors with confirmed diagnosis (PTC or PTMC) and Tx staging excluded, 57 cases (32.76%) were observed infiltration of thyroid capsule (T4a or T4b staging) and 115 cases (46.56%) of 247 assessable data (diagnosis of PTC or PTMC and Nx staging excluded) happened lymph node metastases (N1a or N1b staging). In *RAS*-like groups, however, two (22.22%) of the nine malignant nodules and four (40.00%) of the 10 cases were observed to have infiltration of the thyroid capsule and lymph node metastases, respectively. The dominating aberrations may be the *BRAF*^V 600E^ mutant and deletion-insertions, along with *RET* mutants and fusions.

### Concurrent molecular alterations and histopathological characteristics

**Figure 3:**
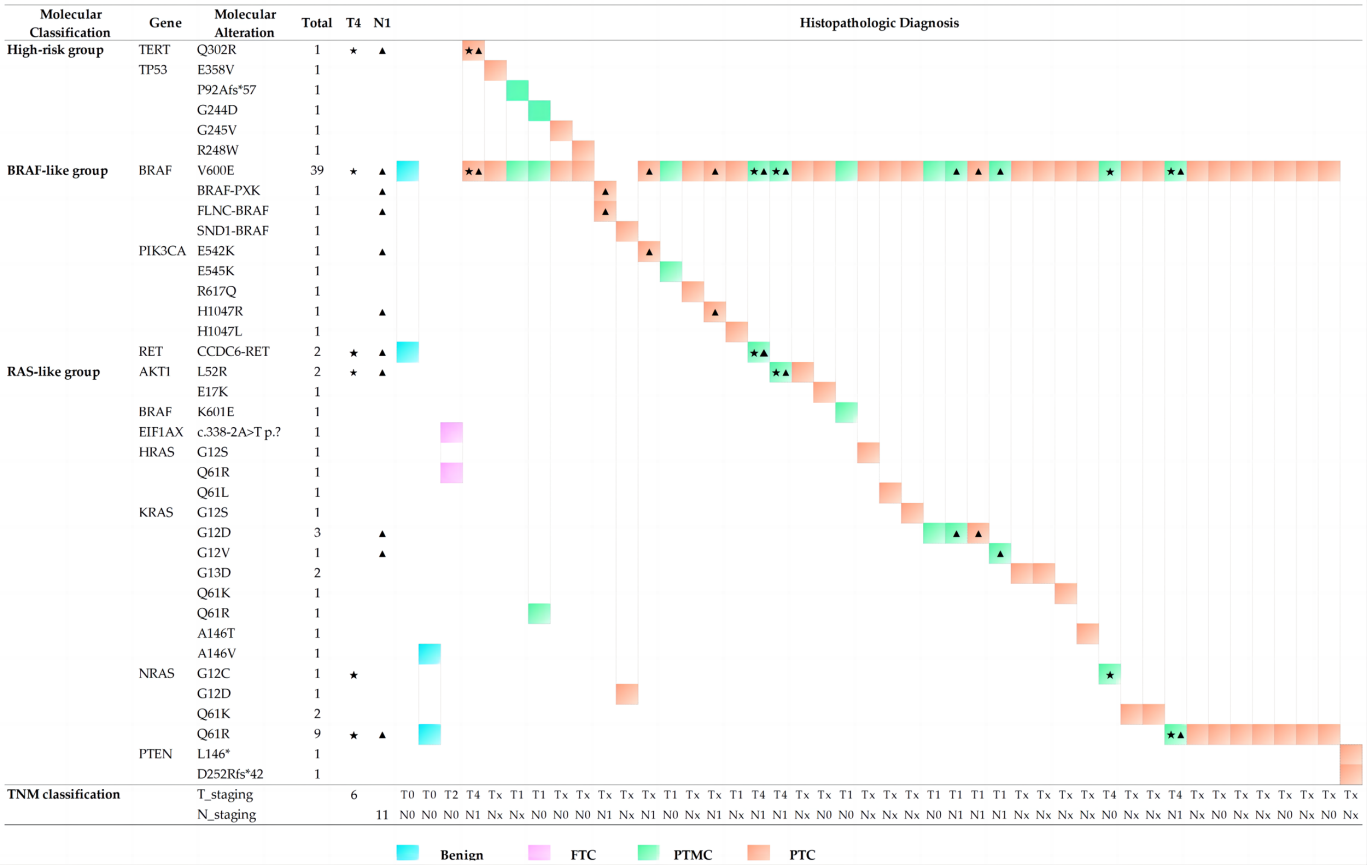
Histopathologic diagnosis and TNM staging in thyroid nodules with concurrent molecular alterations.

A total of 44 nodules with concurrent molecular alterations were isolated and separately analyzed to determine whether some aberrations tended to co-occur with others and present different histopathological characteristics. Figure 3 shows that all cases were classified as PTC or PTMC, with the exception of two benign nodules and one FTC. The most common *BRAF*^V 600E^ could be detected in 39 nodules (88.64%) concurrently with other 25 mutants from eight genes, including *TERT, TP53, PIK3CA, AKT1, BRAF*^K601E^*, HRAS, KRAS,* and *NRAS*, followed by the *NRAS*^Q61R^ mutant, which occurred in nine nodules (20.45%). Among the remaining five cases without the *BRAF*^V 600E^ mutant, two PTC nodules were related to the *BRAF* rearrangements with the *PXK*, *FLNC,* or *SND1* gene, while one PTC nodule underwent double deletions in the *PTEN* gene and one benign nodule revealed concurrent mutants in *KRAS*^A146V^ and *NRAS*^Q61R^. An unknown mutant in the protein detected in *EIF1AX*^c.338−2A>T^ was concurrent with *HRAS*^Q61R^ in a FTC nodule. These findings agreed with previous reports (Fagin & Wells Jr, 2016).

**Figure 4:**
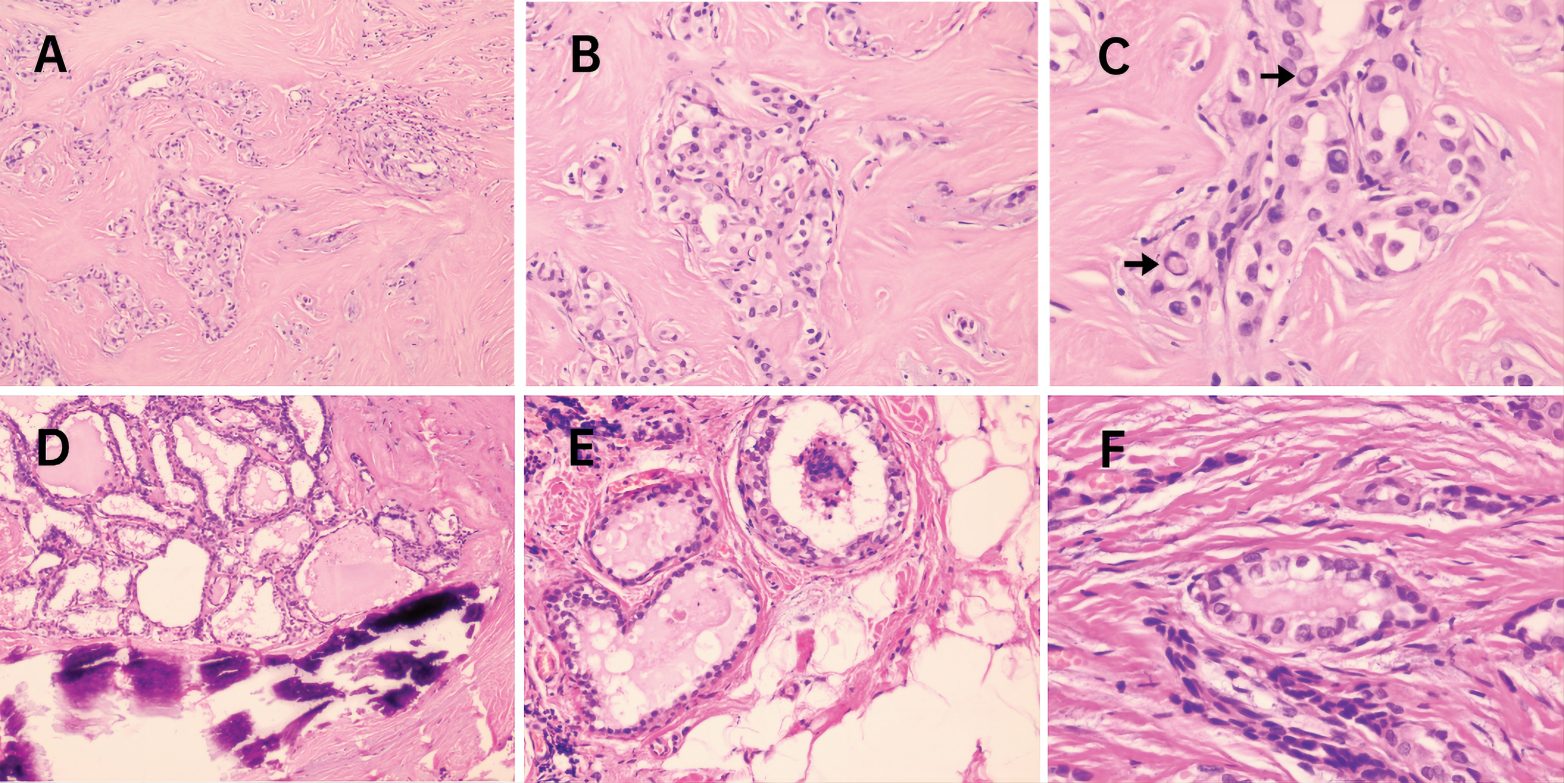
Photomicrographs from 2 cases of FV-PT(M)C with concurrent molecular alterations. In the first case (A–C), molecular testing revealed *BRAF*^V600E^ co-existence with *TP53* ^G244D^ and *KRAS*^Q61R^. Tumor cells arranged in solid nests and showed classical nuclear features of PTC including several intranuclear pseudoinclusions (arrows in C) though being diagnosed as FV-PTMC with poorly differentiated appearance. In the second case (D–F), molecular testing revealed *BRAF*^V600E^ co-existence with *TERT*^Q302R^. The nodule was classified as FV-PTC with calcification, and extrathyroidal extension was present (E) with locally invading adipose tissue. H&E staining. (A and D) 100 × magnification; (B and E) 200 × magnification; (C and F) 400 × magnification. FV-PT(M)C, follicular variant subtype of papillary thyroid carcinoma or microcarcinoma.

Previous studies have focused on the relationship of molecular markers with distant metastases, such as the *BRAF*^V 600E^ mutation and *TERTp* mutations. These were frequently found to co-exist and their presence was considered to be valuable for PTC relapse risk assessment (Ulisse et al., 2021; Soares et al., 2021). Likewise, in our cohort, potentially aggressive nodules may also be related to concurrent molecular alterations involving *BRAF*^V 600E^being accompanied by *TERT*^Q302R^, *AKT1*^L52R^, *NRAS*^G12C^, *NRAS*^Q61R^ and *CCDC6*-*RET* fusion. Five cases with lymph node metastases (N1 staging, marked with triangle symbols in Fig. 3) presented with infiltration of the thyroid capsule (T4 staging, marked with pentagram symbols in Fig. 3) after surgery resection, although one nodule with concurrent mutations of *BRAF*^V 600E^ and *NRAS*^G12C^ was an exception. Histopathologic slides of a resection in two cases of FV-PT(M)C were reviewed and the photomicrographs are presented in Fig. 4. The nodule with the combination mutations of *BRAF*^V 600E^, *TP53*^G244D^ and *KRAS*^Q61R^ were poorly differentiated, while the lesion with concurrent alterations of *BRAF*^V 600E^ and TERT^Q302R^ displayed an extrathyroidal extension with regional nodal involvement and local invasion of the skeletal muscle (data not shown) and adipose tissue (Fig. 4). These observations indicated that thyroid nodules with concurrent molecular alterations may be related with poor differentiation, aggressiveness, and a follicular variant of the PTC subtype.

## Discussion

A multigene panel for cytologically indeterminate thyroid nodules was designed, and molecular results were retrospectively assessed for their performance when compared to a clinical diagnosis. The overall consistency was acceptable with high NPV which made the panel a promising tool to rule out the malignant Bethesda category III and IV nodules. Positive tests in the Bethesda V nodules suggested treatment with a total thyroidectomy instead of a lobectomy. Eleven false-negative and five false-positive cases were found in the cohort. During the analysis of the false negatives, the presence of low-frequency mutations that were not highlighted by the automatic analysis software IGV were checked. However, false negatives may have occurred due to: (1) a molecular misdiagnosis caused by limited tumor volume or an improvable sampling technique and preservation method; (2) molecular alterations in genes that occurred beyond the scope of our panel (namely, the detection of 14 thyroid cancer-related genes and 21 types of gene rearrangements). The false positives may have occurred for a number of reasons, including limited clinical follow-up. Additionally, the extremely high prevalence of cancer in the cohort should be noted for clinical reference of multigene testing in presurgical diagnosis of thyroid nodules.

The most observed gene aberration in our cohort referred to driving somatic genetic alterations in the MAPK pathway, including the *BRAF*^V 600E^ mutant, *RET* aberrations, and *RAS* mutations. Furthermore, several molecular aberrations were observed in thyroid cancers or even solid tumors for the first time. There were some infrequently reported alterations, including *EIF1AX* c.338-2A>T splice site *KIAA1217-RET* fusion, that were considered to be pathogenetic mutations or oncogenic driver genes for malignant tumors, as revealed in previous research (Castagna et al., 2020; Elsherbini et al., 2022; Lee et al., 2016; Song et al., 2022; Davis et al., 2020; Lee et al., 2016). These new findings may support genetic profiling and could be potential therapeutic targets for thyroid cancers.

For the purpose of ancillary diagnosis and outcome prediction, the malignancy risk of thyroid nodules could be stratified based on different molecular aberrations (Soares et al., 2021; Yip et al., 2021; Skaugen et al., 2022). In our study, detected molecular aberrations were categorized into three MRGs: the *BRAF*-like group (presenting low differentiation and predominantly relevant to classical PTC with papillary architecture), the *RAS*-like group (displaying high differentiation, less recurrence, and enriched in thyroid carcinomas with follicular-pattern), and the high-risk group (relevant to poorly clinical outcomes and typically coexisting with other alterations) (Cancer Genome Atlas Research, 2014).

The *BRAF*^V 600E^ and mutated *RAS* were considered to be two mutually exclusive drivers of PTC, possibly suggesting similar or redundant downstream effects, and causing different signaling effects followed by profound phenotypic differences (Cancer Genome Atlas Research, 2014; Ren et al., 2022) However, less attention had been paid to concurrent molecular alterations, conveying a significant adverse prognosis and suggesting treatment *via* a total thyroidectomy of the thyroid nodules (Bose, Sacks & Walts, 2019; Ren et al., 2022; Poller & Glaysher, 2017). In our study, some drivers in the PI3K pathway and tumor suppressor genes manifested a tendency to be concurrent with others, such as *BRAF* rearrangements and deletions, *PTEN* deletions, *TERT*^Q302R^, and mutations of *AKT1*, *EIF1AX*, *TP53,* and *PIK3CA.* As shown in Fig. 3, 26 cases (59.09%) in all 44 nodules with concurrent molecular alterations were detected concomitant *BRAF/RAS* mutation. Besides, the nodules that indicated the presence of *TERT* or *TP53* mutants concurrent with *BRAF*-like mutations presented the follicular variant of the PTC subtype and were indicative of poor differentiation or aggressive features. The more aggressive features and poorer progression of the *BRAF*-like group *versus* the *RAS*-like group were consistent with previous reports (Rossi et al., 2022; Krasner et al., 2019).

Bioinformational tools have found that *BRAF* mutants or fusions were strongly separated from the *H/N/KRAS* mutants and partly overlapped the *RET* fusions in clusters (Cancer Genome Atlas Research, 2014). However, in our cohort, *BRAF*^V 600E^ was found to be a co-mutant with *RAS* and *PIK3CA* mutations, while the *RET* fusions were exclusive with the *BRAF* aberrations. These ambiguous discrepancies may have resulted from an unverified computerized algorithm, racial differences, or insufficient sample sizes, *etc*. The types of mutations in the oncogenes or tumor suppressor genes could also determine the biological behavior of malignant tumors (Kelil et al., 2016). In addition to concurrent alterations and mutation types, diverse mutations in the same genes resulting in variants of identical or adjacent codons might be related to different biologic behaviors. For example, *HRAS*^Q61K^ was detected in one PTC, *HRAS*^Q61L^co-mutant with *BRAF*^V 600E^ in one PTC, while *HRAS*^Q61R^ was detected as a co-mutant with the *EIF1AX* mutant in one FTC; *KRAS*^A146T^ or *NRAS*^Q61K^was a detectable co-mutant with *BRAF*^V 600E^in PTCs, though *KRAS*^A146V^ and *NRAS*^Q61R^ were found to be concurrent in a benign nodule. These assumptions appealed to the further classification of molecular alterations and conjoint analysis with pathological results for clinical reference.

Some limits were present in the study: (1) the limitation presented by the extremely high prevalence of cancer in the cohort should be noted, as well as the low sample size of Bethesda III and IV groups; (2) a well-designed prospective study with informed consent of the patients will be essential for more informative results in the future; (3) the long-term follow-up above five years may be beneficial to further summarize the prediction values for remote metastasis and disease outcomes and to elucidate the outcomes of unresected indeterminate nodules that prospectively underwent analysis of the panel in this study.

## Conclusions

The successful presurgical diagnosis of thyroid nodules is debatable. Although molecular tests such as Thyroseq or Afirma have been demonstrated to have a very good performance, they are not readily available. Treatment facilities are often left to find their own resources to identify those indeterminate nodules that need to be surgically treated. Our study reported the results of a multi-gene panel analysis on cytological samples from indeterminate thyroid nodules and proposed a model for risk stratification that was able to predict cases that should then be referred to surgery. However, the extremely high prevalence of cancer in the studied cohort was a limitation of our study.

##  Supplemental Information

10.7717/peerj.16054/supp-1 Supplemental Information 1 List of multigene testing panel

10.7717/peerj.16054/supp-2 Supplemental Information 2 All diagnosis data in the cohort

## Additional Information and Declarations

### Data Availability

The following information was supplied regarding data availability: The raw measurements are available in the Supplementary Files.
